# Did the COVID-19 pandemic impact antibiotic prescribing patterns among dentists?

**DOI:** 10.1038/s41432-024-01087-3

**Published:** 2024-11-20

**Authors:** Akshani Patel, Satish Kumar

**Affiliations:** Arizona School of Dentistry & Oral Health, A.T. Still University, Mesa, AZ USA

**Keywords:** Antibiotics in dentistry, Infection control in dentistry

## Abstract

**Data sources:**

Patient records from Wits Oral Health Centre were collected over two years (March 2019–March 2021). The records were divided into two groups: pre-COVID-19 (March 2019–March 2020) and COVID-19 (March 2020–March 2021). The total sample size was 698 systematically selected patient records who received antibiotics.

**Study selection:**

A systematic random sampling method was used to select a sample of patients who received prescriptions for antibiotics. Patients were included based on their treatment time and need for antibiotic prescription. Patients whose records were missing information or treated by independent private practitioners for whom records access was not feasible were excluded.

**Data extraction and synthesis:**

The extracted data contained information regarding the patients’ demographics, medical histories, dental condition, dental procedure, antibiotic type, frequency, dosage, duration of use, and prescribers’ disciplines. The information was categorized and captured in Microsoft Excel to analyze and create figures and tables. Analysis was performed using descriptive statistics, chi-squared, and z-tests to compare pre-COVID-19 and COVID-19 prescription patterns in RStudio.

**Results:**

From an initial pool of 44,067 patient consultations, a systematic random sample of 698 records met the eligibility criteria for inclusion in this study. The sample was divided between pre-COVID-19 (n = 350) and COVID-19 (n = 348). A significant increase in antibiotic prescriptions was found during COVID-19 (1571 prescriptions) compared to pre-COVID-19 (1109 prescriptions). The most commonly prescribed antibiotics were amoxicillin and metronidazole. Specifically, amoxicillin was used in most cases before and during COVID-19. The combination of amoxicillin with metronidazole increased from 16.6% pre-COVID-19 to 24.4% COVID-19. It was found that many of the antibiotics prescribed from both periods were not clinically indicated (53.1% pre-COVID-19 and 54.3% COVID-19).

**Conclusions:**

The authors concluded that the COVID-19 pandemic significantly increased antibiotic prescriptions despite fewer in-person consultations. This was likely due to insufficient surgical intervention and reliance on antibiotics to treat dental conditions. The lack of proper antibiotic use raises concerns regarding dental practitioners’ appropriate use of antibiotics. There is an urgent need for improved antibiotic stewardship to prevent the misuse and growing public health issue of antimicrobial resistance.

## A Commentary on

**Pillay L, Rikhotso R E**.

Antibiotic prescribing pattern of oral health practitioners before and during the COVID-19 pandemic at Wits Oral Health Centre. *Br J Oral Maxillofac Surg* 2024; 10.1016/j.bjoms.2024.05.005.

**GRADE Rating**: 

## Commentary

Antibiotic resistance is a global threat to public health due to the overuse and misuse of antibiotics in various healthcare settings, including dentistry. The World Health Organization has determined that antimicrobial resistance could be one of the most significant threats to humans and could result in 10 million deaths each year by 2050 if measures are not taken to implement antibiotic stewardship^1–3^.

Dental practitioners often use antibiotics to manage infections or as prophylaxis before specific dental procedures. However, studies have shown that a significant proportion of these prescriptions are unnecessary or inappropriate, resulting in the broader problem of antibiotic resistance^4,5^. Dentists account for around 10% of all antibiotic prescriptions worldwide, making them significant contributors to the overuse of antibiotics^3^. In countries like the United States, dentists are the top prescribers of antibiotics like clindamycin, primarily for outpatient care^6^. There is considerable concern about overprescription in dentistry for conditions that do not require them. A retrospective cohort study examining antibiotic prescriptions in dental practices from 2011 to 2015 found that more than 80% of prophylactic antibiotic prescriptions were unnecessary and did not adhere to antibiotic use guidelines^5^. This infers that a large portion of antibiotic use does not adhere to the clinical guidelines, resulting in the overuse of antibiotics and the rise of antibiotic-resistant bacterial strains. Additionally, studies have found that antibiotics are prescribed in a precautionary form, even though conservative dental treatments would be more beneficial^7^.

This study aimed to understand the antibiotic prescribing patterns of oral health practitioners before and during the COVID-19 pandemic^8^. In particular, to identify the indications and types of antibiotics prescribed, as well as to compare the prescribing patterns before and during the pandemic. The study revealed that, despite a reduction in the number of patient consultations during the COVID-19 pandemic, there was a notable rise in the number of antibiotics prescribed by oral health practitioners compared to the pre-pandemic period. This increased use of antibiotics may be linked to factors such as reduced access to dental care, patients seeking treatment at more advanced stages of dental issues during the pandemic, and inadequate antibiotic stewardship. The retrospective study design of this study limits the authors’ ability to control the variables that may have influenced the antibiotic prescribing patterns. Additionally, the authors of this study only focused on the data from one oral health center, which may result in the findings not being generalizable to other regions. Finally, not collecting data from private practices may have excluded important data regarding the prescribing patterns during COVID-19. However, the data collection process was comprehensive, and the authors defined the methods and inclusion and exclusion criteria well. The sample size of 698 patients was large enough to allow the authors to draw conclusions regarding the shift in antibiotic prescriptions during COVID-19. Additionally, the authors of this study addressed the issue of antibiotic resistance and antibiotic stewardship and the need for initiatives to increase antibiotic stewardship.

The increased prescription of antibiotics in unnecessary situations shows the importance of implementing guidelines and education to prevent unnecessary prescriptions. Clinical practice guidelines have been developed for pulpal and periapical region-related dental pain as well as intraoral swelling^7^. There are well-established guidelines on antibiotic prophylaxis for patients with prosthetic joints^9^ and appropriate use criteria^10^ as well as for patients undergoing dental procedures and having a risk of infective endocarditis due to underlying cardiac conditions is also well-established^11^. These guidelines aim to promote appropriate use of antibiotics in dentistry.

Antibiotic stewardship programs have been created to promote the responsible use of antibiotics in all healthcare settings, including dentistry^1,2^. These programs aim to ensure that antibiotics are only prescribed when necessary and in appropriate dosages. Antibiotic stewardship programs in dentistry can include prescriber education, clinical decision-making tools, and ongoing surveillance of antibiotic use^6,12^. Despite the progress made in antibiotic stewardship, many challenges remain. First is the need for standardized prescribing guidelines across countries and regions, which could result in consistent antibiotic use^3^. Next, patients often pressure dentists into prescribing antibiotics when unnecessary^4^. Legal repercussions and concerns regarding patient satisfaction may result in dentists feeling obligated to prescribe antibiotics and overuse antibiotics in dental practices^12^. More research needs to be conducted on the factors influencing antibiotic prescribing decisions in dentistry to address these challenges. Shared decision-making between dentists and patients is a crucial aspect of antibiotic stewardship. By involving the patient in the decision-making process and explaining the risks and benefits of antibiotic use, dentists can reduce the demand for antibiotics when they are not indicated^13^.

Practice points

- Oral health practitioners should prioritize definitive, conservative dental treatment for dental infections and only prescribe antibiotics when indicated.
- Implementing antibiotic stewardship using shared decision-making tools and adhering to clinical guidelines for antibiotic use can significantly reduce unnecessary antibiotic use and overcome antimicrobial resistance.
